# A Diet Rich in HUFAs Enhances the Energetic and Immune Response Capacities of Larvae of the Scallop *Argopecten purpuratus*

**DOI:** 10.3390/ani13081416

**Published:** 2023-04-20

**Authors:** Isis Rojas, Claudia B. Cárcamo, Yohana Defranchi, Katherine Jeno, José Rengel, Michael Araya, María Elena Tarnok, Luis Aguilar, Gonzalo Álvarez, Paulina Schmitt, Katherina Brokordt

**Affiliations:** 1 Doctorado en Acuicultura, Programa Cooperativo Universidad de Chile, Pontificia Universidad Católica de Valparaíso, Universidad Católica del Norte, Coquimbo 1781421, Chile; 2 Laboratorio de Fisiología Marina (FIGEMA), Departamento de Acuicultura, Facultad de Ciencias del Mar, Universidad Católica del Norte, Coquimbo 1780000, Chile; 3 Centro de Innovación Acuícola (AquaPacífico), Universidad Católica del Norte, Coquimbo 1781421, Chile; 4 Laboratorio de Producción Primaria, Departamento de Acuicultura, Universidad Católica del Norte, Coquimbo 1781421, Chile; 5 Centro de Investigación y Desarrollo Tecnológico en Algas y otros Recursos Biológicos (CIDTA), Facultad de Ciencias del Mar, Universidad Católica del Norte, Coquimbo 1781421, Chile; 6 Laboratorio de Fotofísica y Espectroscopía Molecular, Instituto de Química, Facultad de Ciencias, Pontificia Universidad Católica de Valparaíso, Valparaíso 2340025, Chile; 7 Laboratorio de Genética e Inmunología Molecular, Instituto de Biología, Facultad de Ciencias, Campus Curauma, Pontificia Universidad Católica de Valparaíso, Valparaíso 2362807, Chile; 8 Departamento de Acuicultura, Facultad de Ciencias del Mar, Campus Guayacán, Universidad Católica del Norte, Coquimbo 1781421, Chile; 9 Centro de Estudios avanzados en Zonas Áridas (CEAZA), Coquimbo 1781421, Chile

**Keywords:** mollusk larval immunity, larval metabolism, larval diet, dietary fatty acids

## Abstract

**Simple Summary:**

Scallop aquaculture depends on hatchery-reared larvae that frequently present mass mortalities due to bacterial infections. Herein, it was demonstrated that the administration of a diet based on microalgae rich in omega 3 increases larval cell membrane fluidity and energy metabolic capacity, which in turn enhances immune capacity and resistance to bacterial infection. Additionally, this diet enhances larval growth and survival; thus, its application would be a promising strategy for improving scallop aquaculture productivity.

**Abstract:**

Massive mortalities in farmed larvae of the scallop *Argopecten purpuratus* have been associated with pathogenic *Vibrio* outbreaks. An energetic trade-off between development-associated demands and immune capacity has been observed. Given that highly unsaturated fatty acids (HUFAs) are essential nutrients for larval development, we evaluated the effect of diets based on microalgae low and high in HUFAs (LH and HH, respectively) on the energetic condition and the immune response of scallop larvae. The results showed that the HH diet increased cellular membrane fluidity in veliger larvae. The routine respiration rate was 64% higher in the HH-fed veligers than in the LH-fed veligers. Additionally, the metabolic capacity tended to be higher in the HH-fed veligers than in the LH-fed veligers after the *Vibrio* challenge. After the challenge, the HH-fed veligers presented higher transcript induction of *ApTLR* (immune receptor) and *ApGlys* (immune effector) genes, and the HH-fed pediveligers presented higher induction of *ApLBP/BPI1* (antimicrobial immune effector) gene, than the LH-fed larvae. Furthermore, the HH-fed veligers controlled total *Vibrio* proliferation (maintaining near basal levels) after the bacterial challenge, while the LH-fed veligers were not able to control this proliferation, which increased three-fold. Finally, the HH-fed larvae showed 20–25% higher growth and survival rates than the LH-fed veligers. Overall, the results indicated that the administration of a HH diet increases cell membrane fluidity and energy metabolic capacity, which in turn enhances immunity and the ability to control *Vibrio* proliferation. The administration of microalgae high in HUFAs would be a promising strategy for improving scallop larval production efficiency.

## 1. Introduction

Farmed scallops represented 20% of the total value (USD) of the net income from mollusk aquaculture worldwide in 2020 [1], and it is expected that bivalve farming will continue to grow in the coming years. However, massive larval mortalities associated with bacterial outbreaks have been reported in several commercial hatcheries of bivalve species [2,3,4,5], including *Argopecten purpuratus* [6,7,8], a scallop of great economic importance for Chile and Peru. The effect of environmental conditions on bacterial outbreak intensity [9] and the effect of nutritional status on larval survival in farming conditions [10,11] have been studied. Nonetheless, despite the role of the immune response in the defense against pathogens, few studies have focused on evaluating the effect of dietary enrichment on the immune capacity of bivalve larvae [12], especially in scallop larvae [13,14].

The molluscan innate immune system, which includes both cellular (mediated by hemocytes) and humoral components, involves energetically expensive processes for combating pathogens [15], such as chemotaxis, pathogen recognition, activation of intracellular signaling cascades, opsonization, and phagocytosis [16,17,18]. Therefore, a trade-off between energy allocation in the immune response and other energy-demanding physiological processes, such as growth, development, and reproduction, has been reported in insects and birds [19,20,21]. This trade-off was also observed in *A. purpuratus*, where a decrease in hemocyte amount and quality after spawning, as well as a reduction in hemocyte energy support for the immune response (mediated by molecular sensors and effectors), were reported [22].

Early development is another critical and energetically expensive process due to the severe physiological and morphological changes during larval growth [23,24]. Accordingly, metabolic demands during critical stages in early development could compete with other energetically costly processes, such as the immune response, increasing the susceptibility to potential pathogens. Few studies have evaluated the metabolic demand imposed by pathogenic bacterial infections in scallop larvae. In this regard, it was recently found that the *A. purpuratus* veliger larval stage has the highest metabolic demand and the lowest mitochondrial concentration and energy production efficiency compared with other early developmental stages [25]. This suggests that the veliger stage would have the lowest capacity to overcome pathogen infections. Indeed, massive mortalities most often occur during the veliger stage of this scallop, from which several pathogenic strains of *Vibrio* bacteria have been isolated and identified [14,26].

Nutritional status directly affects the energetic capacity of an organism [27] and has been shown to positively influence both mollusk larval development and immune response performance [28,29]. Among the nutritional components, lipids are essential for appropriate larval development [30,31]. The incorporation of highly unsaturated fatty acids (HUFAs) in the larval diet of some bivalve mollusks is crucial for growth, development, metamorphosis, and settlement [32,33,34,35]. Mollusks, like other invertebrates, are incapable of synthesizing HUFAs de novo; thus, these fatty acids must be obtained from the diet, which mainly comprises microalgae [36]. Lipids, and especially HUFAs, perform two major functions: (1) eicosapentaenoic (EPA: 20:5-ω3) and docosahexaenoic (DHA: 22:6-ω6) acids are elements of cellular membrane phospholipids (polar lipids) and are involved in preserving membrane integrity, fluidity, and biological functionality; and (2) fatty acids are components of triglycerides, which are a main energy source for metabolism [37]. The triglyceride content in pectinid larvae is a good indicator of embryogenesis, growth, and survival [35,38]. In commercial hatcheries, pectinid larvae are fed microalgal mixes containing different HUFA levels, directly influencing farming production performance [30,39,40]. For example, pectinid larvae fed *Chaetoceros calcitrans* (low in HUFA content) have lower settlement capacity than larvae fed *Isochrysis galbana* T-iso (high in HUFA content), a difference attributable to the microalgal fatty acid content [30,40]. The use of a diet rich in HUFAs in adult *Ruditapes philippinarum* clam fostered the incorporation of n-3 and n-6 fatty acids into the cellular membrane hemocytes and the enhancement of their phagocytic capacity [29]. Nonetheless, to our knowledge, there are no studies relating dietary HUFA content with immune capacity in bivalve larvae.

*A. purpuratus* inhabits the Pacific coast from Peru to Chile, where it is intensively farmed [1]. Juvenile availability depends on natural recruitment, which is unstable and restricted to spring and summer seasons, and on hatchery production, which is affected by bacterial outbreaks [41]. Herein, we evaluated the effect of the application of a diet based on microalgae rich in HUFAs on the energetic condition and the capacity of the immune response of larvae (veliger and pediveliger) and early juveniles. To accomplish this, we designed and applied two microalgal dietary treatments, one with a low HUFA content and the other with a high HUFA content. We compared larvae and early juveniles fed with these diets in terms of (1) the cellular membrane fluidity through fluorescence spectroscopy analysis; (2) the metabolic capacity at basal and post-*Vibrio splendidus* exposure conditions, measured as the activity of key enzymes of the anaerobic (PK) and aerobic metabolism (CS and ETS), as well as the anaerobic capacity (PK:CS) and energy production efficiency (ETS:CS) indices; (3) the metabolic demand (only for veliger), measured as the individual respiration rate basally (routine) and postexposure to a *V. splendidus* pathogen strain; (4) the relative expression of immune-related genes associated with each step of the immune response (sensing, signaling, and effectors) at basal and post-*V. splendidus* exposure conditions; (5) the larval capacity to reduce *Vibrio* proliferation after exposure to a *V. splendidus* pathogen strain; and (6) growth and survival rates. To our knowledge, this is the first study to evaluate the effect of the application of microalgae-based diets with contrasting HUFA levels on the metabolic and immune response capacities under basal and post-bacterial-challenge conditions in scallop larvae. By this means, we aimed to evaluate whether this feeding strategy potentially decreases larval susceptibility to *Vibrio* pathogens, particularly in the most susceptible veliger larval stage, and to support hatchery production.

## 2. Materials and Methods

### 2.1. Ethics Approvals

Animal care was conducted in strict accordance with the recommendations in the CCAC guidelines (http://www.ccac.ca/Documents/Standards/Guidelines URL (accessed on 26-10-2020)). The protocol for sampling procedures and experimental manipulations was approved by the Bioethics Committee of the Universidad Católica del Norte (CEC-UCN/06) and the Chilean National Agency for Investigation and Development (ANID).

### 2.2. Larval Rearing

Adult *Argopecten purpuratus* scallops (*n* = 100, 70–80 mm height) were collected from the aquaculture concession area belonging to the Universidad Católica del Norte at Tongoy Bay (Coquimbo, Chile). Two spawning and larval cultures were made for the purpose of this study. Larvae and juveniles obtained from the first spawn were used to measure the cellular membrane fluidity, the metabolic capacity (via enzymatic activity of PK, CS, and ETS), and the mRNA expression of immune-related genes. The second spawn was carried out for oxygen consumption measurement and capacity to reduce *Vibrio* proliferation at the veliger stage. The spawning protocol was modified from Rojas et al. [14]. Mature scallops were induced to spawn with the addition of concentrated microalgae (*Isochrysis galbana* clone T-iso + *Chaetoceros calcitrans* + *Pavlova lutheri*, 17 × 10^6^ cells mL^−1^). Gamete fertilization was performed in a proportion of 7–10 sperm per oocyte, and the resulting eggs were maintained in tanks with slight aeration. The obtained D larvae (48 h postfecundation, hpf) were transferred to two separated recirculating culture systems (RAS) (modified from [42]). Each RAS consisted of 12 cylindrical tanks filled with filtered (1 µm) and sterilized (UV) 150 L of seawater, maintained at room temperature (17 ± 1 °C) and with continuous aeration. Two dietary treatments, namely, (1) high in HUFA (hereafter named HH) and (2) low in HUFA (hereafter named LH), were applied to each RAS (12 tanks per diet) (Supplementary Figure S1). Larvae were reared in these systems at an initial density of 30 larvae mL^−1^ for 40 days. The assessed stages were veliger (120–130 µm: 8 days postfecundation, dpf), pediveliger (190–210 µm: 21 dpf), and early juvenile (500–600 µm: 40 dpf). The protocol for the second spawning and larval rearing treatments were the same, but the larval culture was carried out until 21 dpf (pediveliger).

### 2.3. Microalgae Cultivation, Proximal Analysis, and Diet Design

#### 2.3.1. Culture Conditions

Two diets were designed to achieve contrasting HUFA contents in larval diets: LH and HH diets. Both diets comprised commercial microalgal species normally used for feeding scallop larvae. Specifically, *Isochrysis galbana* clone T-iso, *Pavlova lutheri*, and *Chaetoceros gracilis* were selected for their high HUFA contents [43,44]. Each microalgal species was subjected to two different nutritional conditions during its culture. F/2 medium [45] (without nutritional modification, NNM) was used to cultivate the microalgal species used in LH treatment. A modified F/2 medium with a phosphorus excess (with nutritional modification, WNM) was used to stimulate lipid biosynthesis in every microalgal species of the HH diet [46] (see below). All microalgae were produced under controlled conditions in a batch system (20 L) with continuous aeration and continuous illumination at 50 µmol photon·m^2^·s^−1^ at 20 ± 1 °C. Microalgae were used in the exponential growth phase.

#### 2.3.2. Proximate Composition

- Carbohydrates: One liter of each microalgal culture was dehydrated at 60 °C until constant mass. The dry mass was pulverized and homogenized with deionized water (1:1 *w*/*v*). The phenol–sulfuric acid method was applied [47] to determine the total carbohydrate concentration. Briefly, 5 mL of 20% trichloroacetic acid was mixed with 1.5 mL of the homogenate, heated at 65 °C for 1 h, and centrifuged at 6000× *g* for 15 min. Then, 1 mL of the supernatant was mixed with 2.5% phenol and concentrated sulfuric acid. The carbohydrate concentration was determined using an EPOCH microplate spectrophotometer (BioTek) at 490 nm, and a 50 µg·mL^−1^ solution of glycogen in deionized water was used as a standard. The carbohydrate content was expressed in µg·mg^−1^ dry mass.
- Proteins: Protein content was quantified following Brokordt et al. [48]. Briefly, a 3 mg wet mass of each microalga was homogenized in a buffer containing 32 mM Tris-HCl at pH 7.5, 2% SDS, 1 mM EDTA, 1 mM Pefabloc, and 1 mM protease inhibitor cocktail (Sigma). The obtained homogenates were incubated at 100 °C for 5 min. A second incubation (for 5 min at 100 °C) was applied after resuspending the homogenate in 100 μL of homogenization buffer. The obtained homogenate was centrifuged for 20 min at 10,600× *g*. A Micro-BCA kit was then used to quantify total protein in an aliquot of the supernatant in an EPOCH microplate spectrophotometer (BioTek) at 660 nm, using a 0.4 mg·mL^−1^ solution of albumin serum in deionized water as a standard.
- Lipids and fatty acid composition: Lipid extraction was performed according to Folch et al.’s [49] protocols. Fatty acid methyl esters (FAMEs) of total lipids were prepared by transmethylation with 14% BF3 MeOH for 10 min at 60 °C. FAMEs were then obtained by liquid–liquid extraction with hexane and washed with 20% NaCl. The organic phase was roto-evaporated, resuspended in 1 mL of hexane, and filtered (0.22 µm PVDF filter). FAMEs were analyzed by injecting 1 µL of the obtained organic phase in a Clarus 600, PerkinElmer gas chromatograph with an FID detector, using a Supelco Wax 320 Omega column (30 m × 0.25 µm film thickness) and helium as the carrier gas. The temperature ramp started at 140 °C for 5 min, and then the temperature increased at a rate of 2 °C per minute until reaching 240 °C and being maintained at that level for 5 min. Individual FAs were quantified by relative percentage by comparing the retention times (RT) and the peak area of the sample with a FAME standard (Sigma, CRM47885, St. Louis, MO, USA).

### 2.4. Diet Design

The nutritional modification (i.e., excess of phosphorous) applied during microalgal cultivation significantly affected their proximal composition, particularly the lipid and fatty acid contents (Supplementary Table S1). The HH and LH diets were designed based on the fatty acid profile analysis obtained for each microalga (Table 1). Different proportions of each microalga from the two culture systems (WNM or NNM) were mixed for each dietary treatment. Microalgae were administrated in one daily ration of 126 × 10^3^ cells mL^−1^, and the feed ration was subsequently adjusted to the larval density every two days [42]. Considering the ontogeny-associated nutritional requirements [23], an initial and a continuation microalgal mix were used. The initial mix was applied from day 2 until day 8 postfecundation and the continuation mix was applied from day 9 until the end of the culture (10 days post-settlement). The initial mix consisted of *I. galbana* and *P. lutheri*, and the continuation mix consisted of *I. galbana*, *P. lutheri,* and *C. calcitrans*. Previous studies have indicated that *Chaetoceros spp*. present low digestion in the early stages [50]; however, they are well-digested and necessary for inducing settlement in advanced stages due to their high EPA contents [51]. The initial LH mix consisted of 70% *I. galbana* and 30% *P. lutheri*, yielding a 1.65 mg HUFA·L mix^−1^. The HH initial mix consisted of equal parts of the same microalgal species (i.e., *I. galbana* and *P. lutheri*), but cultured WNM, yielding a 4.3 mg HUFA·L mix^−1^, with 2.6 times more HUFA than in the LH initial diet. In the continuation diet, 20% *C. gracilis* (WNM for HH diet) was added to each diet. The HH continuation diet had 2.7 times more HUFAs than the continuation LH diet. Regarding macronutrients, the protein content was ~1.5 times higher initially than in the continuation diet, independent of LH or HH treatment. Carbohydrate delivery tended to be higher in the LH diet during the entire cultivation period (1.6 times), while the total lipid content was similar in both dietary treatments.

### 2.5. Membrane Fluidity Analysis in Larvae and Early Juveniles

To evaluate the functional effect of the application of diets with different HUFA contents, the fluidity of cell membranes in larvae and early juveniles fed with the applied diets was evaluated. This analysis involved two biophysical parameters associated with membrane fluidity: (1) The packing order of the acyl chains of the membrane lipids was revealed by DPH (1,6-diphenyl-1,3,5-hexatriene) and TMA-DPH (1-(4-trimethylammonio)-6-phenyl1,3,5-hexatriene) fluorescence anisotropy. The DPH fluorescent probe was inserted in the central zone of the lipid phase of the bilayer [52]. A TMA-DPH fluorescent probe was localized in the vicinity of glycerol and the upper segments of the phospholipid chains [53]. (2) The packing of lipid polar heads and/or the polarity of the environment to the bilayer hydrophobic–hydrophilic interface was evidenced through changes in the Laurdan (6-lauryl-2-dimethylaminonaphthalene) generalized polarization (PG) [54]. Samples of larvae (veliger and pediveliger) and early juveniles fed the two diets were obtained, centrifuged at 4 °C, and immediately frozen at −80 °C until analysis.

#### 2.5.1. Decalcification and Incubation

Larvae (5 mg wet mass) and early juveniles (10 mg wet mass) were diluted in EDTA-PBS (5%) and incubated for 1 h at room temperature with mechanical agitation (~3× *g*). Samples were then washed 3 times in PBS using a centrifuge at 1400× *g* for 5 min and resuspended in 1 mL of PBS. Finally, samples were incubated with 4 µM DPH (1,6-diphenyl-1,3,5-hexatriene), TMA-DPH (1-(4-trimethylammonium)-6-phenyl1,3,5-hexatriene), or Laurdan (6-lauroil-2-dimethylaminonaphthalene) with mechanical agitation (~3× *g*) for 30 min at 30 °C.

#### 2.5.2. Fluorescence Spectroscopy

Veliger and pediveliger larvae were diluted to a final concentration of 1 mg·mL^−1^ in PBS, while early juveniles were diluted to 2 mg·mL^−1^. DPH and TMA-DPH anisotropy were measured in a K2 spectrofluorometer (ISS Inc., Champaign, IL, USA) using a wavelength of 370 nm for excitation, and in the emission, two long-pass filters of 399 nm and 420 nm were used to eliminate scattering. Generalized polarization of Laurdan was measured at 440 and 490 nm (emission) and excited at 375 nm. Laurdan GP values were calculated according to the GP = I_440_ − I_490_/I_440_ + I_490_ equation [54]. All measurements were made at 17 °C.

### 2.6. Bacterial Challenge

A bacterial challenge was carried out with the pathogenic strain VPAP18 of *Vibrio splendidus*, which was isolated from an *A. purpuratus* veliger larvae massive mortality episode in a commercial hatchery [7]. This strain was cultivated in tryptic soy broth (Difco) supplemented with 2% NaCl medium at 22 °C overnight in a mechanical shaker (~2× *g*). A sublethal infection dose was applied based on Rojas et al. [7] and Rojas et al. [14], which considered the relative larval/juvenile concentration per flask to be infected in each ontogenic stage. Therefore, the veliger stage (120 µm) was exposed to a bacterial dose of 210 colony-forming units (cfu) per larvae, pediveligers (210 µm) were exposed to 620 cfu·larvae^−1^, and early juveniles (500 µm) were exposed to 3500 cfu·individual^−1^.

For every developmental stage, individuals were obtained from the 12 tanks, pooled (according to each diet), redistributed in 24 experimental units (1 L glass flask; 12 per diet) for the bacterial challenge, and maintained in a thermoregulated bath at 17 °C (hatchery rearing temperature) with slight aeration (using air-stone diffusers to maintain the dissolved oxygen level above 80%) (Supplementary Figure S1). The larval density per flask varied according to the developmental stage following Rojas et al. [14]: 35,000 larvae·flask^−1^ for veligers, 25,000 larvae·flask^−1^ for pediveligers, and 500 ind·flask^−1^ for early juveniles. For each dietary treatment, half of the flasks were challenged with *V. splendidus* VPAP18 (as described above) for 24 h, and the other half were maintained undisturbed (control). Six experimental units were added for the veliger stage group (both challenged and undisturbed) to quantify *Vibrio* proliferation. Then, larvae or juveniles from each flask were sieved and split into two Eppendorf tubes (1.5 mL). One tube was immediately stored at −80 °C until the enzyme analyses, and the larvae from the other tube were mixed with TRIzol^®^ reagent (1:10), incubated for 12 h at 4 °C, and then stored at −80 °C until the immune gene expression analyses.

### 2.7. Enzymatic Assays

#### 2.7.1. Tissue Homogenization

Larvae were weighed and homogenized in a buffer (1:5 *w*/*v*) containing 0.1% Tween 20, 2 mM EDTA-Na2, 5 mM EGTA, 150 mM KCl, 1 mM dithiothreitol, and 50 mM imidazole-HCl. The homogenates were centrifuged at 600× *g* for 10 min at 4 °C, and the supernatants were immediately used for enzymatic assays. All assays were run in duplicate at controlled room temperature (17 °C) using an EPOCH spectrophotometer (Biotek, Winooski, VT, USA), and the specific activities were expressed in international units (mmol of substrate converted to product per min) per mg of wet mass.

#### 2.7.2. Pyruvate Kinase (PK)

PK activity was measured following Brokordt et al. [55]. Briefly, the supernatant obtained after homogenization was diluted in a reaction buffer (1:5 *v*/*v*) containing 5 IU of lactate dehydrogenase, 5 mM ADP, 0.2 mM NADH, and 5 mM phosphoenolpyruvate (omitted for the control), with a pH of 6.6. Enzyme activity was measured following the absorbance changes of NADH at 340 nm with a molar extinction coefficient of 6.22 mM^−1^ cm^−1^.

#### 2.7.3. Citrate Synthase (CS)

CS activity was measured following Brokordt et al. [55]. The supernatant obtained after homogenization was diluted in a reaction buffer (1:3 *v*/*v*) containing 75 mM TRIS-HCl, 0.3 mM DNTB (5,5-dithio-bis-2-nitrobenzoic acid), 0.2 mM acetyl Co-A, and 0.3 mM oxaloacetate (omitted for the control), with a pH of 8.0. Enzyme activity was measured following the absorbance changes at 412 nm associated with the transfer of sulfhydryl groups from CoASH to DTNB, with a molar extinction of 13.6 mM^−1^ cm^−1^.

The CS-specific activity was expressed in international units (IU) per mg of wet mass. The PK:CS ratio was used as an indicator of aerobic to anaerobic capacity [56].

#### 2.7.4. Electron Transport System (ETS)

The ETS activity assay followed Packard’s [57] methodology with minor modifications. The supernatant obtained after homogenization was diluted in a buffer (1:10 *v*/*v)* containing 75 mM TRIS-HCl, 5% polyvinylpyrrolidone (PVP), 153 mM MgSO4, and 0.1 % Tween 20. Enzyme activity was measured using a reaction mix containing 75 mM TRIS, 0.1% Tween-20, 1.7 mM NADH, 0.25 mM NADPH, and 0.2% iodonitrotetrazoliumodium (INT). Absorbance changes were measured at 490 nm to follow the extinction of INT for 10 min. The molar extinction used for INT was 15.9 mM^−1^ cm^−1^.

The ETS:CS ratio was used as a proxy of mitochondrial ATP production efficiency given that CS is considered an indicator of mitochondrial content [58].

### 2.8. Oxygen Consumption

Respiration rates (RRs) were measured in veliger larvae from the second spawn. Two-hour postchallenged larvae (and their controls) from each diet treatment were transferred to 2 mL glass vials equipped with oxygen sensor spots, where RR was determined at a density of ~35 larvae·mL^−1^. Measurements were performed using a 24-channel oxygen meter (SDR^®^, PreSens GmBh, Regensburg, Germany) at controlled room temperature (17 °C). PreSens Measurement Studio software was used to determine the O_2_ concentration every 15 s. Eight replicates of each treatment (LH-control, LH-challenged, HH-control, HH-challenged) were registered, and two blanks (SSW and SSW + VPAP18) with four replicates each were measured to obtain the background and bacterial respiration.

A linear regression was used to calculate the RR by plotting O_2_ saturation as a function of time, standardized by the larval number and corrected by the blanks. Only values ≥ 65% saturation were considered to avoid any possible (differential) influence of low dissolved O_2_ on the RR of the animals. RR was expressed as the individual-specific rate (nmol O_2_·h^−1^·larvae^−1^).

### 2.9. RNA Isolation, cDNA Synthesis, and Relative mRNA Expression of Immune-Associated Genes by RT–qPCR

TRIzol Reagent (Thermo Scientific, Waltham, MA, USA) was used for the extraction of total RNA following the manufacturer’s instructions. Concentration and purity were examined by spectrophotometry using an EPOCH spectrophotometer (Biotek, Winooski, VT, USA), and the integrity was assessed by formaldehyde/agarose gel electrophoresis. cDNA was obtained using 0.8 µg of total RNA and a PrimeScript™ RT Reagent Kit with gDNA Eraser (Takara Bio Inc., Shiga, Japan) following the manufacturer’s instructions.

Transcriptional levels of five genes associated with different steps of the immune response (i.e., receptor, transcriptional regulator, and antimicrobial effectors) previously characterized for *A. purpuratus* adults were evaluated in larvae and early juveniles: a Toll-like receptor (*ApTLR*¸ GenBank MH732641), an inhibitor of the NF-kB (*ApIkB*, GenBank FJ824733), and three immune effectors—a big defensin (*ApBD1*, GenBank KU499992), an LPS-binding/bactericidal-permeability-increasing protein (*ApLBP/BPI1*, GenBank MN295978), and a g-type lysozyme (*ApGLys*, GenBank AY788903). β-actin (*ApBactin*, GenBank FE895980.1) was used as an endogenous control to normalize the experimental results [59]. The primers used in this study and previously validated for *A. purpuratus* are listed in Supplementary Table S3.

RT–qPCR was used to measure the expression of immune-related genes using an Mx3000P Real-Time PCR System (Agilent Technologies, Santa Clara, CA, USA). RT–qPCR was performed using a 20 µL reaction volume containing 2 µL of cDNA, 10 µL of Takyon Low ROX SYBR 2X MasterMix blue dTTP (Eurogentec, Seraing, Belgium), and 0.6 µL of each primer (10 mM). Samples were tested in triplicate. The initial denaturation time was 10 min at 95 °C, followed by 40 PCR cycles of 95 °C for 15 s, and 60 °C for 1 min. After the PCR cycles, the purity of the PCR product was checked by the analysis of its melting curve; the thermal profile for melting curve analysis consisted of denaturation for 15 s at 95 °C, lowering to 55 °C for 15 s, and then increasing to 95 °C for 15 s with continuous fluorescence readings. The comparative ΔΔCt method [60] was used to estimate the relative mRNA expression of each studied gene.

### 2.10. Postchallenge Vibrio Quantification

To evaluate the effect of LH or HH diet on the control of *Vibrio* proliferation, total *Vibrio* count was quantified in veliger larvae 24 h post-VPAP18 challenge (and not challenged controls). This quantification was performed only in the veliger larvae because this stage presented the highest mass mortalities due to vibriosis. The challenged veligers (and controls) were filtered with a 100 µm sieve and then washed 2 times with sterile seawater (SSW: microfiltered at 0.22 µm and sterilized). The obtained larval wet mass (50 ± 5 mg) was diluted in SSW (1:10 *w*/*v*), homogenized, and vortexed for 10 s. The homogenate was diluted in SSW (1:10,000 *v*/*v*) and then homogenized again for 10 s. This second homogenate (50 µL) was cultivated in a TCBS agar plate at 22 °C for 24 h. The total colony-forming units (cfu) present in each plate were then quantified. The total number of cfu was standardized with respect to the wet weight of the homogenized larvae, and an inoculum of the microalgae mixture used to feed the larvae was used as a control.

### 2.11. Growth and Survival

The estimation of growth and survival rates was carried out throughout the larval culture for each dietary treatment. In this second culture, larval density was adjusted according to larval mortality in each dietary treatment; however, due to a technical problem, this culture was stopped after 21 dpf. Survival was estimated in five samples of 1 mL taken from each tank as the percentage difference of larval density with respect to initial stocking density. From each sample, the size of 20 larvae was measured in photographs taken at 100× magnification using image analysis software (ImageJ-FIJI).

### 2.12. Statistical Analysis

The results are presented as the mean and standard error of the mean (SE). ANOVAs were applied when data met normality as tested with Shapiro–Wilks and homoscedasticity as indicated by the Fligner–Killeen test. When the results did not meet these requirements, a robust ANOVA for trimmed means was applied [61]. Tukey’s HSD *post-hoc* test was applied to test specific differences [62]. The results were considered significantly different at *p* < 0.05. All statistical analyses were conducted with R CRAN using the “WR2S” package for robust ANOVA and “agricolae” for *post-hoc* tests.

## 3. Results

### 3.1. Membrane Fluidity Analyses

**Figure 1:**
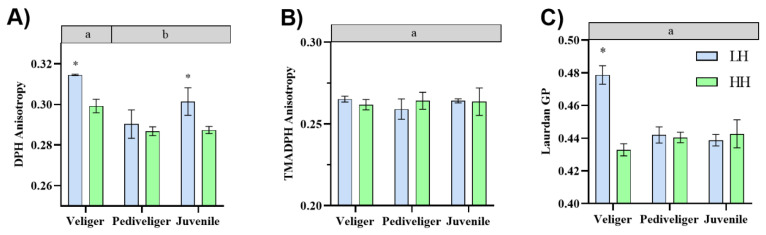
Effect of diets low (LH) and high (HH) in HUFAs on the fluidity of cellular membranes of larvae and juveniles of the scallop *Argopecten purpuratus*. Cell membrane fluidity was measured through the anisotropy of (**A**) DPH (1,6-diphenyl-1,3,5-hexatriene) and (**B**) TMA-DPH (1-(4-trimethylammonium)-6-phenyl1,3,5-hexatriene), and by (**C**) Laurdan PG (generalized polarization) using fluorescence spectroscopy analysis. Different lowercase letters show significant differences among developmental stages (*p* < 0.05). Asterisks (*) indicate significant differences between dietary treatments within each developmental stage (*p* < 0.05); *n* = 3 pools per condition.

Among the parameters associated with cell membrane fluidity, the DPH anisotropy (packing order of acyl tails) was significantly affected by the developmental stage and the diet; the Laurdan GP was significantly affected by both factors and their interaction; and TMA-DPH anisotropy was affected by neither of these factors (Figure 1; ANOVA, Supplementary Table S4). Among the assessed stages, veliger larvae displayed the lowest membrane fluidity regardless of the applied diet, as shown by the highest DPH anisotropy (Figure 1A). On the other hand, veliger larvae and juveniles fed the HH diet showed the lowest DPH anisotropy and thus the highest membrane fluidity. Additionally, veliger larvae fed the HH diet presented lower Laurdan GP than those fed the LH diet and thus a higher membrane fluidity (Figure 1C).

### 3.2. Enzyme Activity

#### 3.2.1. Pyruvate Kinase (PK)

**Figure 2:**
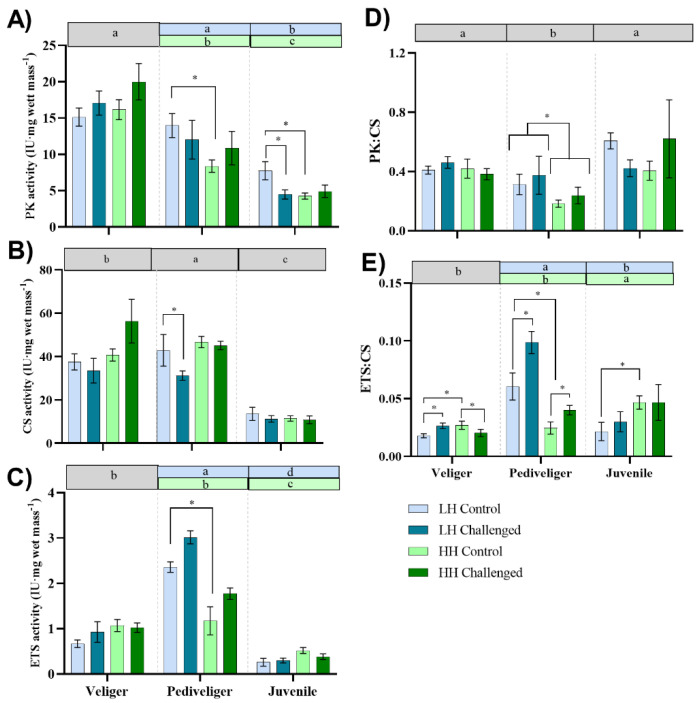
Effect of diets low (LH) and high (HH) in HUFAs on the energy metabolism of larvae and juveniles of the scallop *Argopecten purpuratus* under unchallenged (control) and *Vibrio splendidus* (VPA18)-challenged conditions. Energy metabolism was measured via the activity of key metabolic enzymes and their ratios: (**A**) pyruvate kinase (PK), (**B**) citrate synthase (CS), and (**C**) electron transport system (ETS) activities; and (**D**) PK:CS (anaerobic to aerobic capacity) and (**E**) ETS:CS (an indicator of mitochondrial efficiency) ratios. Different lowercase letters show significant differences among developmental stages (gray bars) or diet (blue/green bars) (*p* < 0.05). Asterisks (*) indicate significant differences between treatments within each developmental stage (*p* < 0.05); *n* = 6 pools per condition.

The PK activity varied at the basal (control) level during the early ontogeny of the scallop, with the highest activity in the veliger stage and the lowest activity in the early juvenile stage despite the dietary treatment. Pediveligers and early juveniles fed the LH diet showed higher PK activity than the HH-fed larvae and juveniles (Figure 2A; ANOVA, Supplementary Table S5). Within each developmental stage, the analyses showed no significant effects of the bacterial challenge and the diet on PK activity for veliger and pediveliger larvae (ANOVA, Supplementary Table S6). Nevertheless, the PK activity was significantly affected by the interaction of the diet and the bacterial challenge in early juveniles. The LH-fed juveniles showed a decrease in PK activity after bacterial exposure, and the HH-fed juveniles showed lower PK activity under both basal and postchallenge conditions. Interestingly, both veliger and pediveliger larvae that were fed the HH diet tended to exhibit increased PK activity after the *V. splendidus* challenge (Figure 2A).

#### 3.2.2. Citrate Synthase (CS)

The CS basal activity significantly varied during early ontogeny regardless of the applied diet, and it was higher in the veliger and pediveliger stages than in juveniles (Figure 2B; ANOVA, Supplementary Table S5). Within each developmental stage, the analyses showed that CS activity was affected by the diet and the bacterial challenge only in pediveliger larvae (ANOVA, Supplementary Table S6). CS activity was higher in pediveligers fed the HH diet at both basal and postchallenge levels; however, CS decreased after the bacterial challenge in pediveliger fed the LH diet. Interestingly, CS activity tended to increase in the HH-fed veliger larvae after the challenge (Figure 2B).

#### 3.2.3. Electro Transport System (ETS)

The ETS basal activity significantly varied throughout early ontogeny and with the interaction between ontogeny and diet. The LH-fed pediveliger larvae showed the highest ETS level (Figure 2C; ANOVA, Supplementary Table S5). Within each developmental stage, the analyses showed that the ETS activity was not affected by the experimental conditions in the veliger stage; however, the HH-fed larvae tended to have higher ETS activity than the LH-fed larvae regardless of the bacterial challenge. For pediveliger larvae, the ETS activity was affected by the bacterial challenge and the diet but not by the interaction between these factors. Both basal and postchallenge ETS levels were higher in the LH-fed larvae than in the HH-fed larvae, and they were not different from the postchallenge HH-fed pediveligers (ANOVA, Supplementary Table S6). In juveniles, the ETS activity was affected by the diet only, with those fed the HH diet showing the highest activity.

#### 3.2.4. PK:CS

The PK:CS ratio was significantly affected by the developmental stage and the diet. Pediveliger larvae showed the lowest PK:CS basal ratio, and larvae and juveniles fed the LH diet showed higher PK:CS ratios than those fed the HH diet (Figure 2D; ANOVA, Supplementary Table S5). The analyses made within each developmental stage revealed that only pediveliger larvae were affected by the diet, where those fed the LH diet showed a higher PK:CS ratio than those fed the HH diet (ANOVA, Supplementary Table S6).

#### 3.2.5. ETS:CS

The ETS:CS ratio was significantly affected by the developmental stage, the diet, and the interaction between these factors (Figure 2E; ANOVA, Supplementary Table S5). The basal ETS:CS ratios were higher in LH pediveligers and in HH juveniles. The within-developmental-stage analyses revealed that this parameter was affected by the interaction between the diet and the bacterial challenge at the veliger stage (ANOVA, Supplementary Table S6), with the HH-fed veligers showing higher basal ETS:CS than the LH-fed veligers. This ratio increased after the bacterial challenge in the LH-fed veligers and decreased in the HH-fed veligers. At the pediveliger stage, both the diet and the challenge affected the ETS:CS ratio. The bacterial challenge caused an increase in the ETS:CS ratio regardless of the dietary treatment, although this ratio was higher in the LH-fed pediveligers. Last, in early juveniles, only the diet affected the ETS:CS ratio, where those fed the HH diet showed the highest ratio.

### 3.3. Respiration Rate

**Figure 3:**
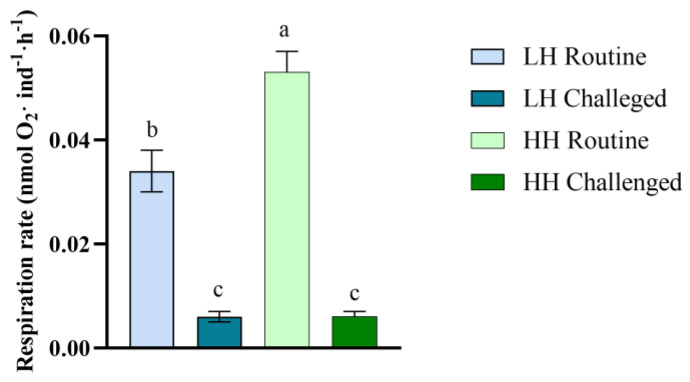
Effect of diets with low (LH) and high (HH) HUFAs on the respiration rate of veliger larvae of the scallop *Argopecten purpuratus* under unchallenged (routine) and *Vibrio splendidus* (VPA18)-challenged conditions. Different lowercase letters indicate significant differences between treatments (*p* < 0.05); *n* = 6 pools per condition.

The metabolic capacity measured through enzymatic activities was complemented with the respiration rate, measured as the individual oxygen consumption rate in the veliger larvae. The respiration rate was significantly affected by the interaction between the diet and the bacterial challenge (Figure 3; ANOVA, Supplementary Table S7). Veliger larvae fed the HH diet presented higher routine respiration rates than those fed the LH diet. However, after the bacterial challenge, both the LH- and HH-fed veligers exhibited drastically decreased respiration rates.

### 3.4. Expression of Immune-Related Genes

**Figure 4:**
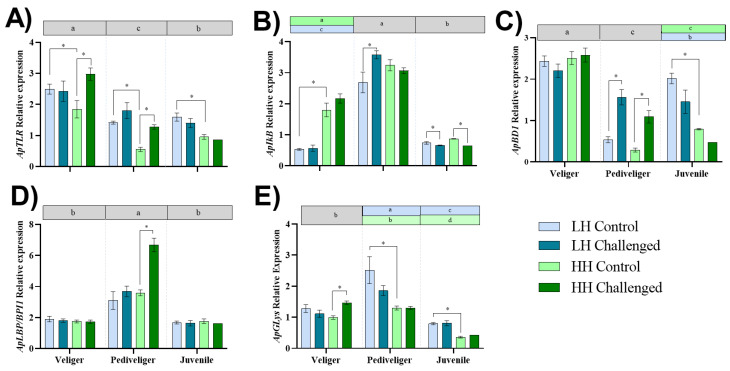
Effect of diets with low (LH) and high (HH) HUFAs on the expression of immune-related genes in larvae and juveniles of the scallop *Argopecten purpuratus* under unchallenged and *Vibrio splendidus* (VPA18)-challenged conditions. (**A**) Toll-like receptor (*ApTLR*), (**B**) inhibitor of NF-kB (*ApIkB*), (**C**) big defensin antimicrobial peptide (*ApBD1*), (**D**) LPS-binding/bacterial permanently increasing protein (*ApLBP/BPI1*), and (**E**) G-type lysozyme (*ApGLys*). Different lowercase letters show significant differences among developmental stages (gray bars) or diet (blue/green bars) (*p* < 0.05). Asterisks (*) indicate significant differences between treatments within each developmental stage (*p* < 0.05); *n* = 6 pools per condition.

The basal expression of all the analyzed immune-related genes changed through the ontogenic stages, and most of them were also affected by the dietary treatment (Figure 4; ANOVA, Supplementary Table S8). The relative expression of the recognition receptor *ApTLR* was highest in the veliger larvae and lowest in the pediveliger larvae, and was higher in the LH-fed larvae than in the HH-fed larvae at the basal level (Figure 4A). The analyses within each developmental stage showed a significant interaction between the diet and the bacterial challenge at the veliger stage. *ApTLR* was overexpressed after bacterial challenge only in the HH-fed veliger larvae (Figure 4A; ANOVA, Supplementary Table S9). A similar result was observed in pediveliger larvae, where postchallenge *ApTLR* overexpression was observed only in the HH-fed larvae. In early juveniles, only the diet affected *ApTLR* expression, where those fed the LH diet showed the highest expression.

The basal *ApIkB* expression throughout early ontogeny was affected by the developmental stage, the diet, and the interaction between these factors (Figure 4B; ANOVA, Supplementary Table S8). The pediveliger larval stage presented the highest basal *ApIkB* expression regardless of the diet type, followed by the HH-fed veliger larvae and the LH-fed veligers and juveniles, which presented the lowest expression. The within-stage analyses revealed that only the diet affected *ApIkB* expression at the veliger stage, and those fed the HH diet showed the highest expression. The interaction between diet and immune challenge affected *ApIkB* expression in pediveliger larvae. The LH-fed larvae overexpressed this gene after the challenge, and the HH-fed pediveligers maintained high basal and postchallenge levels, similar to the LH-fed pediveligers. *ApIkB* expression was only affected by the bacterial challenge in early juveniles, and its expression was decreased after bacterial exposure in both the LH- and HH-fed juveniles.

The basal expression of the antimicrobial peptide *ApBD1* was affected by the developmental stage and its interaction with the dietary treatment (Figure 4C; ANOVA, Supplementary Table S8). The veliger larvae exhibited the highest basal expression, while the pediveligers showed the lowest *ApBD1* expression. However, basal *ApBD1* was higher in the LH-fed juveniles than in the HH-fed juveniles. The within-stage analyses showed that *ApBD1* expression was not affected by the contrasting factors at the veliger stage, but pediveligers were affected by both diet and bacterial challenge, and juveniles were only affected by the diet (ANOVA, Supplementary Table S9). *ApBD1* expression increased significantly after bacterial challenge in both the LH- and HH-fed pediveligers, attaining similar levels (Figure 4C). The LH-fed juveniles showed higher *ApBD1* expression than the HH-fed individuals at both basal and postchallenge levels.

The basal expression of the antimicrobial protein *ApLBP/BPI1* varied during early ontogeny due only to the developmental stage, with the pediveliger larvae showing the highest expression regardless of the diet (Figure 4D; ANOVA, Supplementary Table S8). The analyses within each developmental stage showed that *ApLBP/BPI1* expression was not affected by the contrasting factors at the veliger and juvenile stages. Nonetheless, pediveligers were affected by both diet and bacterial challenge (ANOVA, Supplementary Table S9). *ApLBP/BPI1* expression increased significantly after the bacterial challenge only in the HH-fed pediveligers, whereas the LH-fed pediveligers maintained their expression after the bacterial challenge (Figure 4D).

The basal expression of the antimicrobial effector *ApGLys* varied during early ontogeny due to the developmental stage and the diet but was not affected by the interaction of these factors (Figure 4E; ANOVA, Supplementary Table S8). The LH-fed pediveliger larvae showed the highest basal expression, followed by the veliger larvae and the HH-fed pediveliger larvae. The lowest basal level was observed in the HH-fed juveniles. Within-developmental-stage analyses revealed that only the veligers fed the HH diet significantly overexpressed *ApGLys* after the bacterial challenge (ANOVA, Supplementary Table S9). *ApGLys* expression was affected only by diet in the juveniles, with those fed the LH diet showing higher expression than those fed the HH diet regardless of the bacterial challenge.

### 3.5. Vibrio Proliferation

**Figure 5:**
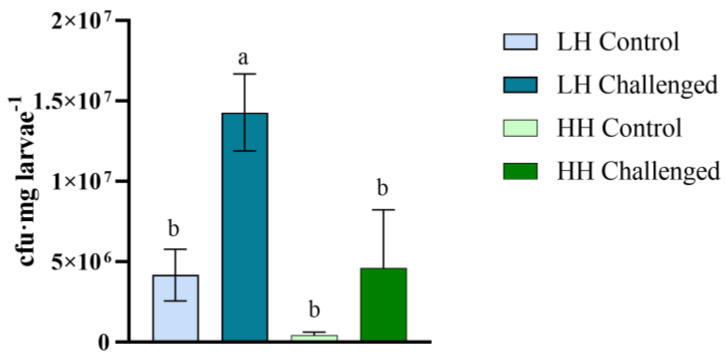
Total *Vibrio* colony-forming unit (cfu) counts in *Argopecten purpuratus* veliger larvae fed diets low (LH) and high (HH) in HUFAs and under unchallenged (control) and challenged with *Vibrio splendidus* (VPAP18) conditions. The lowercase letter indicates significant differences among treatments (*p* < 0.05); *n* = 3 pools per condition.

The capacity to control *Vibrio* proliferation at the basal level and after bacterial challenge with VPAP18 was evaluated in veliger larvae fed the LH and HH diets. Both the diet and the bacterial challenge affected the number of *Vibrio* colony-forming units (cfu) in the veliger larvae (ANOVA, Supplementary Table S10). The unchallenged larvae showed lower cfu content than the postchallenge veligers, with a tendency to be higher in LH larvae at the basal level. However, after the VPAP18 challenge, *Vibrio* cfu levels were significantly lower in the HH-fed veligers than in the LH-fed veligers (Figure 5). The control TCBS agar plates inoculated with each microalgae diet used for veliger feeding did not present *Vibrio* growth.

### 3.6. Growth and Survival

**Figure 6:**
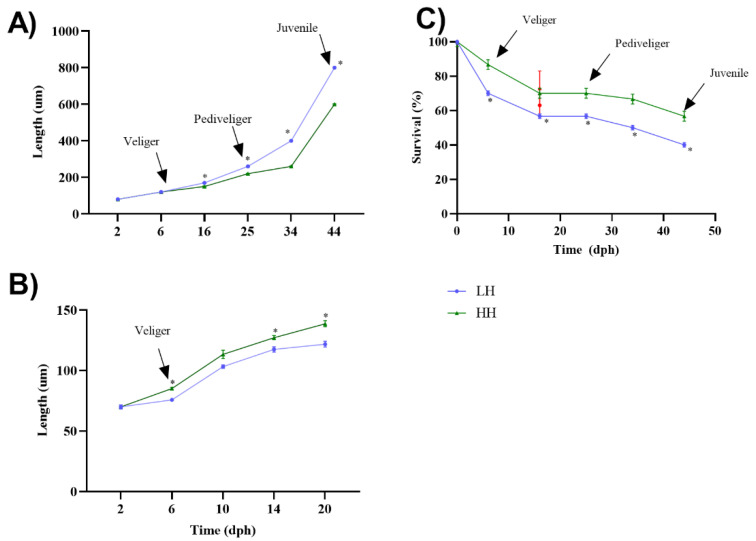
Growth and survival rates of *Argopecten purpuratus* larvae and early juveniles fed diets low (LH) and high (HH) in HUFAs. Growth rates are shown for two cultures; (**A**) the density was not adjusted and (**B**) the density was adjusted according to the differential survival rate observed in each dietary treatment. The survival rate (**C**) for both cultures is presented in the same graph (second culture indicated by the red line). Asterisks (*) indicate significant differences between dietary treatments at each developmental stage (*p* < 0.05).

Growth was measured in larval cultures from two independent spawnings, but only in the second spawning event was the density adjusted according to the differential survival rate observed in each dietary treatment. In each culture, the applied diet affected the larval size and survival in interaction with the time of culture (ANOVA, Supplementary Table S10). During the first culture experiment, the LH-fed individuals were larger than the HH-fed individuals from Day 16 (late veliger) until Day 44 at the early juvenile stage (Figure 6A). However, during the second culture (where density was adjusted), the larval sizes were larger (22–25%) in the HH-fed larvae than in the LH-fed larvae from Day 6 (early veliger) until Day 20 (late veliger), when this culture was stopped due to technical problems (Figure 6B). On the other hand, the survival rate was consistently higher (20–25%) in larvae (or early juveniles) fed the HH diet than in those fed the LH diet in both cultures (Figure 6C).

## 4. Discussion

In aquaculture, the formulation of specific diets normally seeks to increase growth and survival in the different stages of organism development. This improvement is especially critical in the early developmental stages, as in a short period of time there are important changes in the metabolic demands associated with the morphogenesis process. Additionally, during early ontogeny, massive mortalities caused by *Vibrio* pathogens have been observed in *Argopecten purpuratus* hatcheries, mainly at the veliger stage [41]. Previous findings suggest that the higher susceptibility of veliger larvae to bacterial infections would be associated with a lower metabolic capacity to support the immune response [14,25]. The results obtained in the present study indicate that the administration of a diet rich in highly unsaturated fatty acids (HUFAs) (an essential component in the diet of bivalve mollusks [33,63]) to scallop larvae increases cell membrane fluidity and energy metabolic capacity, and enhances the immune response capacity and ability to control *Vibrio* proliferation.

### 4.1. Effect of Dietary HUFA Levels on Cellular Membrane Fluidity during Early Development

One of the aspects that could improve the incorporation of HUFAs in diets is the fluidity of cell membranes. Thus, the effect of feeding with microalgae with contrasting levels of HUFAs on larval membrane fluidity was evaluated first in the present study.

The cell membrane fluidity was measured through DPH, TMA-DPH, and Laurdan PG anisotropy by fluorescence spectroscopy analysis. A lower DPH anisotropy and Laurdan generalized polarization (indicative of a modification in both the fatty acid hydrophobic tails and phospholipid head polar groups of cell membranes) were found in veliger larvae fed the high in HUFAs (HH) diet compared with those fed the diet low in HUFAs (LH), indicating higher membrane fluidity. Consistently, the HH-fed juveniles also presented a higher fluidity in the fatty acid lipid tails but not in the hydrophilic layer, as shown by the lower DPH anisotropy. Conversely, anisotropy parameters were not affected by dietary treatments in the pediveliger larvae. Together, these results suggest that dietary HUFA levels contained in microalgae-based diets affect the membrane composition in some stages of the early development of *A. purpuratus*, directly altering its fluidity. In agreement with these results, a recent study using lipidomic analyses reported that dietary fatty acids influence cellular membrane fluidity in mussel larvae and juveniles [64]. It has also been reported that lipid content variation in *A. purpuratus* adults, specifically phosphatidylcholine, cholesterol, and triglycerides, is influenced by dietary lipids [65], with both cholesterol and triglycerides affecting membrane fluidity [65].

The increase in membrane fluidity would be associated with an improvement in the efficiency of membrane protein activity, with higher metabolic rates in cells with more polyunsaturated membranes (reviewed by [27]). Interestingly, our results show that the veliger larvae fed the HH diet, along with presenting the highest levels of membrane fluidity, presented higher routine metabolic rates than the LH-fed veligers. This could facilitate metabolic support of vital activities, including the immune response capacity, as shown in mouse macrophages, which increased their phagocytic capacity after having been fed a diet rich in HUFAs [66].

Cell membrane fluidity was not different between the pediveliger larvae fed the HH and LH diets. Triglycerides instead of membrane lipids have been proposed as a main energy source for *A. purpuratus* pediveliger larvae [65]. Additionally, HUFAs such as EPA and ARA are used as energy sources [67], but DHA, which plays mainly a structural role (i.e., cellular membranes), was the main HUFA in the HH diet supplied in the present study. On the other hand, less complex lipids, such as monounsaturated (MFA) and saturated (SFA) fatty acids, are important energy sources for scallop larvae [30,68], especially palmitic (16:0) and palmitoleic acid (16:1), because they are more assimilable than HUFAs through the β-oxidation pathway [69]. Although the total lipid content was similar between the supplied HH and LH diets, the SFA content was higher in the HH diet, suggesting a potential higher energy availability for larvae fed this diet. Additionally, a high SFA diet content has been associated with a greater adhesion capacity to bacterial films in mouse macrophages, suggesting a positive effect on the immune response capacity [66].

### 4.2. Effect of Dietary HUFA Levels on Energy Metabolism during Early Development and Bacterial Challenge

Energy metabolism was measured throughout early ontogeny via key metabolic enzymes and their ratios. The results showed that, regardless of the applied diet, glycolytic anaerobic support of energy metabolism is more important in the early (veliger larvae) than in late developmental stages (juveniles) at basal levels, as indicated by the PK activity. The anaerobic capacity could be related to a higher natatory demand in early larval stages adapted to planktonic habits [70]. Interestingly, aerobic support of energy metabolism was higher in the pediveliger stage (CS and ETS activities) and more efficient in the LH-fed pediveligers (ETS:CS ratios). On the other hand, as previously found [25], veliger larvae showed low aerobic energy efficiency, but this was improved in the HH-fed veligers. Indeed, during larval culture, a greater natatory capacity was observed in the HH-fed veliger larvae than in the LH-fed veliger larvae. In general, the early juveniles showed the lowest enzyme activities, which can be associated with the change from planktonic (highly active) to benthonic (more sedentary) lifestyles [71]. Nevertheless, the juveniles fed the HH diet showed a higher aerobic energy efficiency than the LH-fed juveniles.

While no changes were observed in the anaerobic and aerobic energy support in the LH-fed veligers after the bacterial challenge, a clear tendency to increase associated enzyme activities (PK and CS) was observed in the HH-fed veligers. Furthermore, an increase in energy production efficiency via aerobic metabolism was observed in both the LH- and HH-fed pediveligers. The observed increase in the activity of key enzymes could be associated with a metabolic strategy to compensate for the reduction in respiration rates after bacterial challenge. In this sense, the HH-fed veligers and pediveligers were capable of compensating by both the glycolytic anaerobic and aerobic pathways, likely via an increase in mitochondrial abundance (as indicated by CS levels). On the other hand, the LH-fed larvae compensated for the postchallenge reduction in the respiration rate by increasing electron transport activity (i.e., mitochondrial efficiency) and thus only by the aerobic pathway. Indeed, previous findings suggest that stressful conditions suppress anaerobic energy metabolism [72], which could explain the LH-fed larvae’s low capacity to augment PK activity.

Although these results shed some light on the metabolic support to overcome a bacterial challenge in scallop larvae, further research is needed to elucidate this aspect. Along this line, it was recently found that after exposure to a bacterial pathogen, there is a strong activation of many biological processes associated with lipid metabolism (including some HUFAs) in *A. purpuratus* veliger larvae, suggesting that the mobilization of these energy molecules supports the immune response [73]. This finding reinforces the idea of the energetic cost of the immune response and the proposed strategy to improve larval immune capacity via HUFA enrichment of their diet. However, the efficiency of the energy support by the lipid pathways should be further explored.

### 4.3. Effect of Dietary HUFAs on the Expression of Immune-Related Genes in Larvae and Early Juveniles

A recent study indicated that both front-loading and efficient induction of immune genes contribute to bacterial pathogen resistance in scallop *A. purpuratus* larvae [73]. Thus, herein, we evaluated the effect of dietary HUFA levels on both the basal (i.e., front-loading) and postchallenge expression of key immune genes previously characterized for *A. purpuratus* [14,74]. Selected genes are associated with pathogen recognition (*ApTLR*), transcriptional regulation (*ApIkB*), and antimicrobial effectors (antimicrobial peptides and proteins: *ApBD1*, *ApLBP/BPI1*, and *ApGlys*). The results showed that the basal expression of all the immune-related genes changed with the ontogenic stage, and most of them also changed with diet; however, the changes depended on the gene, without a consistent pattern among them. Interestingly, contrary to previous findings [14], veliger larvae showed higher basal levels of *ApTLR* and *ApBD1* than pediveligers and juveniles, which suggests that dietary treatment alters basal immune-related gene expression.

The effect of dietary treatment on the capacity to induce immune genes after a bacterial challenge was different for each developmental stage. The fact that the veliger larvae fed the HH diet presented the highest postchallenge induction of *ApTLRs* and *ApGlys* and were able to maintain high basal *ApIkB* and *ApBD1* levels constitutes an improvement in the immune response at this larval stage. Additionally, pediveligers fed the HH diet were capable of inducing *ApTLR* and *ApBD1* but at a similar level to the LH-fed pediveligers, which implies that dietary HUFAs apparently did not affect the expression of these genes. However, only the HH-fed pediveligers were able to induce the antimicrobial protein *ApLBP/BPI1*. Toll-like receptors (TLRs) are membrane-anchored proteins that recognize a wide range of MAMPs/PAMPs and initiate the activation of intracellular signaling cascades, regulating the transcription of a wide range of immune genes [75]. The enhanced postchallenge induction of this receptor in the HH-fed veligers could be associated with the higher cellular membrane fluidity observed in these larvae, which could confer an increased efficiency to proteins that are embedded in these membranes. Indeed, TLRs initiate downstream signaling via the Rel/NF-kB signaling pathway [76], and it has been suggested that *ApIkB* controls the expression of big defensin (*ApBD1*) via the regulation of Rel/NF-kB in *A. purpuratus* [77]. Thus, the observed higher postchallenge *ApTLR* levels in the HH-fed veligers are consistent with the high expression levels of the associated signaling (*ApIkB*) and antimicrobial effector (*ApBD1* and *ApGlys*) genes observed in these larvae.

Overall, these results suggest that the application of a diet rich in HUFAs enhances the immune response capacity of veliger and pediveliger scallop larvae, including the capacity to recognize, regulate, and induce antimicrobial effectors that control pathogen infection.

### 4.4. Effect of Dietary HUFAs on Vibrio Proliferation in Challenged Veliger Larvae

The capacity to reduce *Vibrio* proliferation after exposure to the *V. splendidus* VPAP18 pathogen strain was then compared in veligers fed the HH and LH diets. The results revealed that although basal total *Vibrio* cfu counts were similar between the HH- and LH-fed larvae, after the VPAP18 challenge, total *Vibrio* proliferation in LH veligers was three-fold higher than in HH larvae. In addition, the proliferation of total *Vibrio* in the HH-fed larvae was not different from the basal *Vibrio* cfu count. These results suggest that dietary HH enhances the capacity of veliger larvae to control *Vibrio* proliferation after exposure to pathogenic bacteria.

The outcome of infectious diseases depends in part on the capacity of the host to control pathogens [78], and this ability is strongly associated with the immune capacity of scallop larvae [73]. Thus, the higher immune response capacity observed in the HH-fed veligers, especially at the level of pathogen recognition and antimicrobial-associated molecules, could be the basis of the observed enhanced ability to control *Vibrio* proliferation after a pathogen challenge. Indeed, lysozyme functions as a crucial biodefense effector against the invasion of bacterial pathogens [79,80], and some studies have demonstrated that molecules could augment the activity of antimicrobial peptides (AMPs) through a synergistic mechanism [81,82], which would be consistent with the observed results.

### 4.5. Effect of Dietary HUFA Levels on Growth and Survival during Early Development

Finally, growth and survival performances were compared between cultures fed with contrasting HUFA levels. Survival was significantly higher (20–25%) in larvae and early juveniles fed the HH diet; thus, in the first culture, density-dependent growth was observed, where the LH diet showed higher growth. High larval density implies less food availability, increased larval collision probability (mechanical stress), higher metabolic rates, and higher metabolite accumulation [83,84]. Thus, in the second culture, density was corrected according to observed survival rates, and as a result, the HH-fed larvae showed higher growth than the LH-fed larvae. Similar results have been reported for blue mussels, for which the application of a lipid-rich diet was associated with higher growth at the juvenile stage [85].

Lipids, particularly those with high contents of HUFAs, are essential components of the diet for scallops during early ontogeny; thus, these results are consistent with those of previous studies [33,51,65,86]. However, here, for the first time, commercial microalgae used for feeding scallop larvae were improved in their HUFA content through nutritional modifications during culture [46]. Indeed, this strategy allowed an almost three-fold increase in total HUFAs, mainly DHA, which, due to their role in maintaining the structural and functional integrity of biological membranes, facilitates rapid conformational changes in membrane proteins and growth-associated processes [87,88].

The intensive culture of scallop larvae in hatcheries involves exposure to various stressors that could decrease culture survival and yield. The greater survival observed in larvae fed the HH diet could be associated with greater availability of energy to tolerate these stress levels. For example, *A. purpuratus* larvae with a higher content of HUFAs presented a greater capacity to induce the anti-stress protein HSP70 after exposure to culture manipulations [89]. On the other hand, greater antioxidant and anti-inflammatory capacities have been associated with feeding mice diets rich in DHA [90]. These and other aspects could be the basis of the higher survival rates in larvae and early juveniles fed the HH diet observed in this study.

## 5. Conclusions

The administration of a diet based on microalgae high in HUFAs during scallop early ontogeny increased cell membrane fluidity and energy metabolic capacity, which in turn enhanced immune capacity and resistance to bacterial infection. Finally, given the additional enhancement of growth and survival, the administration of microalgae with high HUFA content would be a promising strategy for improving scallop larval production efficiency.

## Figures and Tables

**Table 1:** Proximate composition and fatty acid content in the microalgae used for each diet (low and high in HUFAs) in the initial and continuation culture periods of *Argopecten purpuratus* larvae and early juveniles. Fatty acids and HUFAs are expressed in mg·L wet mass^−1^.

	Initial Diet (2–8 dpf)				Continuation Diet (from 9 dpf)					
	Low HUFA		High HUFA		Low HUFA			High HUFA		
Microalgae	*T-iso*	*P. lutheri*	*T-iso* *	*P. lutheri* *	*T-iso*	*P. lutheri*	*C. gracilis*	*T-iso* *	*P. lutheri* *	*C. gracilis* *
**Microalgae proportion**	0.7	0.3	0.5	0.5	0.5	0.3	0.2	0.4	0.4	0.2
Proteins	778.09	297.27	783.45	779.78	555.78	297.27	NC	626.76	623.82	NC
Carbohydrates	63.602	6.885	24.585	33.15	45.43	6.885	NC	19.668	26.52	
Lipids (% dry mass)	14.021	7.161	12.175	14.575	10.015	7.161	0.784	9.74	11.66	0.822
FAME (mg)	7.84	3.267	18.45	8.45	5.6	3.267	0.498	14.76	6.76	0.82
∑ Saturated fatty acid (mg)	3.451	1.533	8.705	3.895	2.465	1.533	0.256	6.964	3.116	0.406
∑ Monounsaturated fatty acid (mg)	1.512	0.69	4.865	2.405	1.08	0.69	0.186	3.892	1.924	0.296
∑ Polyunsaturated fatty acid (mg)	2.66	1.05	4.95	2.2	1.9	1.05	0.056	3.96	1.76	0.118
**HUFAs**										
ARA (mg)	NC		NC				0.0136			0.0016
EPA (mg)	0.051	0.021	0.040	0.0315	0.036	0.021	0.032	0.0324	0.025	0.072
DHA (mg)	1.125	0.452	2.961	1.271	0.804	0.452	0.008	2.368	1.016	0.1
**Total content in the diet mix**										
Proteins	633.8		781.6		367. 1			500.2		
Carbohydrates	46.59		28.87		24.78			18.48		
Lipids (% dry mass)	11.96		13.38		7.156			8.56		
Total ARA (mg)	NC		NC		0.013			0.001		
Total EPA (mg)	0.072		0.072		0.090			0.130		
Total DHA (mg)	1.578		4.232		1.260			3.486		
**Total HUFAs** (**mg**)	**1.650**		**4.304**		**1.363**			**3.617**		
EPA/DHA	0.046		0.017		0.071			0.037		
ARA/EPA	-		-		0.152			0.012		

* Microalgae cultivated with nutritional modification. Protein and carbohydrates are expressed in µg·mg^−1^ wet mass. Content determination was made based on microalgae content in 1 L.

## Data Availability

All datasets generated and/or analyzed in the present study are available from the corresponding author upon request.

## Supplementary Materials

The following supporting information can be downloaded at https://www.mdpi.com/article/10.3390/ani13081416/s1, Figure S1. Experimental design applied to larvae and early juveniles of the scallop *Argopecten purpuratus*. Larvae and juveniles were grown with diets based on microalgae high and low in HUFAs and then exposed to the pathogenic *Vibrio splendidus* strain VPAP18 for 24 h at the veliger (8 dpf), pediveliger (21 dpf), and early juvenile (40 dpf) stages. Table S1. Proximal composition and fatty acid profile of microalgal species used in scallop diets cultivated with or without nutritional modification (WNM and NNM, respectively). Lowercase letters indicate significant differences (*p* < 0.05) between every microalga species. Table S2. T-test comparing proximal composition and fatty acid profile in each microalga used for dietary treatment and cultured with or without nutritional modification. Significant differences (*p* < 0.05) are in bold. Table S3. Nucleotide sequences of primers for RT–qPCR used in this study. Table S4. Two-way ANOVA (A) and robust two-way ANOVA (B) evaluating the effect of ontogenic stage and dietary treatment on DPH and TMADPH (A), and Laurdan PG (B) in larvae and early juveniles of the scallop *A. purpuratus*. Significant differences (*p* < 0.05) are in bold. Table S5. Two-way ANOVA evaluating the effect of ontogenic stage and dietary treatment on the PK, CS, and ETS activities and the PK:CS and ETS:CS ratios in larvae and early juveniles of the scallop *A. purpuratus*. Significant differences (*p* < 0.05) are in bold. Table S6. Two-way ANOVA evaluating the effect of bacterial challenge with *V. splendidus* VPAP18 and dietary treatment on the PK, CS, and ETS enzyme activities and the PK:CS and ETS:CS ratios in larvae and early juveniles of the scallop *A. purpuratus*. Significant differences (*p* < 0.05) are in bold. Table S7. Two-way ANOVA evaluating the effect of diet and bacterial challenge on the respiration rate (nmol O2·h^−1^·ind^−1^) in veliger larvae of the scallop *A. purpuratus*. Table S8. Two-way ANOVA evaluating the effect of ontogenic stage on the basal relative mARN expression of *ApTLR, ApIkB, ApBD1, ApLBP/BPI,* and *ApGLys* in larvae and early juveniles of the scallop *A. purpuratus*. Significant differences (*p* < 0.05) are in bold. Table S9. Two-way ANOVA evaluating the effect of dietary treatment and bacterial exposure to *V. splendidus* VPAP18 on the relative expression of immune-related genes (*ApTLR, ApIkB, ApBD1, ApLBP/BPI1*, and *ApGLys*) in larvae and early juveniles of the scallop *A. purpuratus*. Significant differences (*p* < 0.05) are in bold. Table S10. Two-way ANOVA evaluating the effect of dietary treatment and bacterial exposure to *V. splendidus* VPAP18 on total *Vibrio* growth in veliger larvae of the scallop *A. purpuratus.* Significant differences (*p* < 0.05) are in bold. Table S11. Two-way ANOVA evaluating the effect of farming time and dietary treatment on *A. purpuratus* larval growth/length and survival. Significant differences (*p* < 0.05) are in bold. References [91,92] are cited in the supplementary materials.
