# Supplementary material for: A Diet Rich in HUFAs Enhances the Energetic and Immune Response Capacities of Larvae of the Scallop Argopecten purpuratus
Source: Animals (Basel). 2023 Apr 20;13(8):1416. doi: 10.3390/ani13081416 (PMC10135034; doi:10.3390/ani13081416)
Supplement: Supplementary file 1 [file animals-13-01416-s001.zip › animals-2225689-supplementary.pdf]

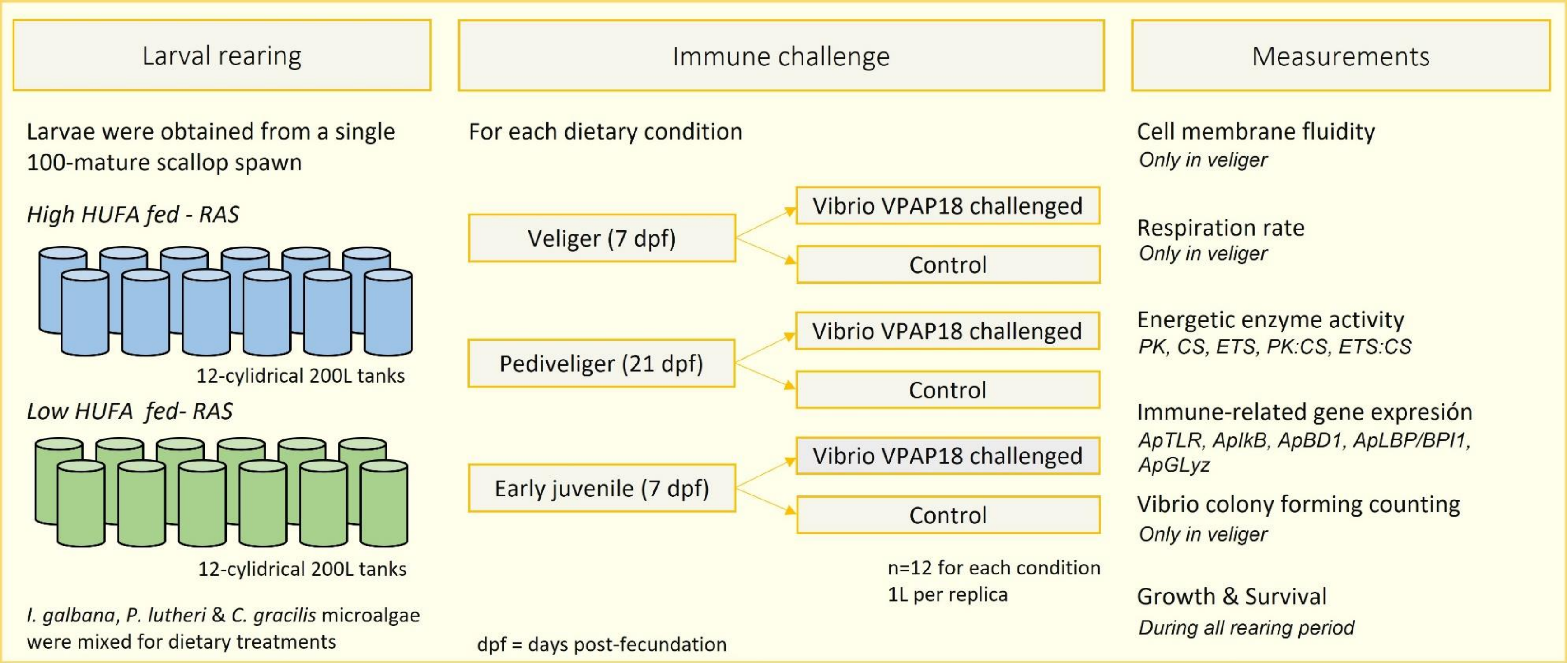

Supplementary Figure S1. Experimental design applied to larvae and early juveniles of the scallop *Argopecten purpuratus*. Larvae and juveniles were grown with diets based on microalgae high and low in HUFAs and then exposed to the pathogenic *Vibrio splendidus* strain VPAP18 for 24 h at the veliger (8 dpf), pediveliger (21 dpf) and early juvenile (40 dpf) stages.

Supplementary Table S1. Proximal composition and fatty acid profile of microalgal species used in scallop diets cultivated with or without nutritional stress (WNM and NNM, respectively). Lowercase letters indicate significant differences (P< 0.05) between every microalga species.

Microalgae specie	*I. galbana clon T-iso*		*P. lutheri*		*C. gracilis*	
Culture method	NNM	WNM	NNM	WNM	NNM	WNM
Proteins (ug. mg wet mass^{-1})	1111.56 (75.34)[b]	1566.9 (107.55)[a]	990.9 (38.51)[b]	1559.56 (136.16)[a]	NC	NC
Carbohydrates (ug. mg wet mass^{-1})	90.86 (4.36)[a]	49.17 (9.64)[b]	22.95 (4.14)[b]	66.33 (7.48)[a]	NC	NC
Lipids (% dry mass)	20.03	24.35	23.87	29.15	3.92	4.11
FAME (mg)	11.2	36.9	10.89	16.9	2.49	4.1
∑ Saturated fatty acid	4.93 (0.21)[b]	17.41 (0.33)[a]	5.11 (0.08)[b]	7.79 (0.08)[a]	1.28 (0.006)[b]	2.03 (0.01)[a]
∑ Monounsaturated fatty acid	2.16 (0.22)[b]	9.73 (0.20)[a]	2.3 (0.003)[b]	4.81 (0.03)[a]	0.93 (0.001)[b]	1.48 (0.004)[a]
∑ Polyunsaturated fatty acid	3.8 (0.12)[b]	9.9 (0.12)[a]	3.5 (0.08)[b]	4.4 (0.04)[a]	0.28 (0.008)[b]	0.59 (0.01)[a]
**Saturated fatty acids**						
C14: Myristic acid	2.990 (0.227)[b]	9.324 (0.439)[a]	3.092 (0.062)[b]	4.162 (0.015)[a]	0.606 (0.014)[b]	0.761 (0.009)[a]
C15:0 Pentadecylic acid	0.03584 (0.00)[b]	0.175 (0.009)[a]	0.034 (0.003)[a]	0.043(0.0008)[a]	0.023 (0.001)	0.035 (0.008)
C16:0 Palmitic acid	1.850 (0.015)[b]	7.481 (0.101)[a]	1.920 (0.025)[b]	3.419 (0.024)[a]	0.593 (0.004)[a]	1.108 (0.003)[b]
C18:0 Stearic acid	0.054 (0.002)[b]	0.381 (0.0)[a]	0.051 (0.002)[b]	0.119 (0.0)[a]	0.054 (0.0009)[b]	0.118 (0.001)[a]
**Monounsaturated fatty acids**						
C14:1 Myristoleic acid	0.026 (0.0)	0.059 (0.003)	0.029 (0.001)	0.018 (0.0)	0.007 (0.0)	0.009 (0.0006)
C16:1 Palmitoleic acid	0.542 (0.003)[b]	1.623 (0.003)[a]	0.571 (0.020)[a]	0.547 (0.006)[a]	0.893 (0.001)[a]	0.675 (0.006)[b]
C18:1n9c Oleic acid	1.591 (0.232)[b]	8.016 (0.204)[a]	1.694 (0.015)[b]	4.214 (0.032)[a]	0.028 (0.0002)[a]	0.015 (0.0005)[b]
**Polyunsaturated fatty acids**						
C18:2n6c Linoleic acid	0.715 (0.033)[b]	0.926 (0.01)[a]	0.713 (0.003)[a]	0.550 (0.006)[b]	0.025 (0.008)[b]	0.064 (0.006)[a]
C18:3n3 Linolenic acid	1.302 (0.056)[b]	2.909 (0.06)[a]	0.986 (0.014)[b]	1.180 (0.01)[a]	NC	0.009 (0.006)

C18:3n6 G-linolenic acid	0.094 (0.003)	NC	0.21 (0.001)^{a}	0.038 (0.001)^{b}	0.014 (0.008)	0.019 (0.005)
C20:4n6 Arachidonic acid (ARA)	NC	NC	NC	NC	0.068 (0.009)	0.008 (0.009)
C20:5n3 Eicosapentaenoic acid (EPA)	0.073 (0.006)	0.081 (0.003)	0.07 (0.005)	0.063 (0.0008)	0.161 (0.023)^{b}	0.363 (0.012)^{a}
C22:6n3 Docosahexaenoic acid (DHA)	1.608 (0.02)^{b}	5.922 (0.04)^{a}	1.509 (0.06)^{b}	2.542 (0.04)^{a}	0.014 (0.002)	0.050 (0.011)

ANOVAs followed by a Tukey test were used to compare statistical differences between NNM and WNM treatments for each microalga specie.

Supplementary Table S2. T test comparing proximal composition and fatty acid profile in each microalga used for dietary treatment and cultured with or without nutritional stress. Significant differences (P< 0.05) are in bold.

	I. galbana clon T-iso			*P. lutheri*			*C. gracilis*		
Factor	**t**	**DF**	***P***	**t**	**DF**	***P***	**t**	**DF**	***P***
Proteins (ug. mg wet mass^{-1})	3.469	3.575	**0.03**	4.019	2.318	**0.044**			
Carbohydrates (ug. mg wet mass^{-1})	3.938	2.78	**0.033**	5.074	3.121	**0.013**			
Lipids (% dry mass)									
FAME (mg)									
∑ Saturated fatty acid	31.76	1.69	**0.002**	32.22	1.021	**0.018**	54.68	1.598	**0.001**
∑ Monounsaturated fatty acid	29.3	1.564	**0.003**	62.35	1.01	**0.009**	116.3	1.217	**0.002**
∑ Polyunsaturated fatty acid	33.45	2	**0.0009**	8.984	1.489	**0.028**	17.09	1.494	**0.01**
**Saturated fatty acids**									
C14: Myristic acid	12.81	1.501	**0.016**	16.62	1.123	**0.027**	8.941	1.725	**0.019**
C15:0 Pentadecylic acid	14.68	1	**0.043**	1	1.855	0.429	6.063	1.125	0.085
C16:0 Palmitic acid	54.88	1.04	**0.009**	42.81	1.999	**0.0005**	84.3	1.79	**0.0003**
C18:0 Stearic acid	19.1	1.028	**0.03**	27.4	1	**0.023**	35.78	1.742	**0.001**
**Monounsaturated fatty acids**									
C14:1 Myristoleic acid	8	1	0.079	5.5	1	0.114	3	1	0.204
C16:1 Palmitoleic acid	203.3	1.965	**<0.0001**	0.552	1.16	0.668	33.07	1.047	**0.016**
C18:1n9c Oleic acid	20.75	1.968	**0.025**	69.11	1.42	**0.001**	18.38	2	**0.002**
**Polyunsaturated fatty acids**									
C18:2n6c Linoleic acid	5.591	2.000	**0.030**	20.220	1.590	**0.006**	18.380	2.000	**0.002**
C18:3n3 Linolenic acid	19.490	1.988	**0.002**	10.630	1.865	0.110	4.500	1.000	0.139

C18:3n6 G-linolenic acid	23.750	1.000	**0.026**	68.600	1.855	**0.000**	2.357	2.000	0.142
C20:4n6 Arachidonic acid (ARA)							2.343	1.908	0.149
C20:5n3 Eicosapentaenoic acid (EPA)	0.868	1.590	0.496	1.268	1.017	0.422	38.100	1.965	**0.0008**
C22:6n3 Docosahexaenoic acid (DHA)	81.640	1.702	**0.001**	12.990	1.877	**0.007**	12.330	1.000	0.051

T-tests were used to compare statistical differences between NNM and WNM treatments for each microalga specie.

Supplementary Table S3. Nucleotide sequences of primers for RT–qPCR used in this study.

Gene	GenBank number	Primer sequence (5′-3′)	Reference
ApTLR	MH732641	**F** CGACAAAACAGAGAAACAAATGGC<br>**R** GTGAACCTCAGTCCGTCAATCT	Brokordt et al., 2019 [22]
ApIkB	FJ824733	**F** GCGTTGATGGTGTATGGTAC<br>**R** TCTGCCGTAATTCGTCGTTG	Oyanedel *et al.*, 2016 [77]
ApBD1	KU499992	**F** CTCGTCCCTCCCTAGTAAGATG<br>**R** GCACTTGTAACTTCCACAAACG	González *et al.*, 2017 [91]
ApLBP/BPI1	MN295978	**F** CTGCTGCCAACCGTTCTGC<br>**R** CGCATGTGCAGATCAACCTGG	González et al., 2020 [74]
ApGLys	AY788903	**F** GGAGACCATCACCATGCTTACG<br>**R** GGGAAATATGTGCGCAGCTGTC	González *et al.*, 2022 [92]
Apβactin	FE895980.1	**F** GAATCTGGCCCATCCATTGT<br>**R** CGTTCTCGTGGATTTTTTTCAAGT	Coba *et al.*, 2016 [59]

Supplementary Table S4. Two-way ANOVA (A) and robust two-way ANOVA (B) evaluating the effect of ontogenic stage and dietary treatment on DPH and TMADPH (A) and Laurdan PG (B) in larvae and early juveniles of the scallop *A. purpuratus*. Significant differences (P< 0.05) are in bold.

**Two-way ANOVA (A)**						
**Parameter**	**Source**	**DF**	**SS**	**MS**	**F**	**P**
DPH	Stage	2	0.0007159	0.0003579	37.799	**4.18×10^{-5}**
	Diet	1	0.0006717	0.0006717	70.936	**1.46×10^{-5}**
	Stage×Diet	2	0.0000191	0.0000095	1.008	0.403
	Residual	9	0.0000852	0.0000095		
TMADPH	Stage	2	0.00001	8.97×10^{-6}	0.079	0.924
	Diet	1	0.00001	1.33×10^{-5}	0.117	0.738
	Stage×Diet	2	0.00005	2.57×10^{-5}	0.227	0.801
	Residual	11	0.00121	1.13×10^{-4}		

**ROBUST two-way ANOVA (B)**			
**Parameter**	**Source**	**Value**	**P**
Laurdan PG	Stage	12.1823	**0.038**
	Diet	11.3590	**0.009**
	Stage×Diet	29.9682	**0.003**

Supplementary Table S5. Two-way ANOVA evaluating the effect of ontogenic stage and dietary treatment on the PK, CS, and ETS activities and the PK:CS and ETS:CS ratios in larvae and early juveniles of the scallop *A. purpuratus*. Significant differences (P< 0.05) are in bold.

Factor	Source	DF	SS	MS	F	*P*
PK	Stage	2	12.7	6.355	37.991	$\mathbf{2.64\times10^{-8}}$
	Diet	1	1.38	1.382	8.261	**0.00815**
	Stage×Diet	2	1.36	0.681	4.073	**0.02943**
	Residual	25	4.18	0.167		
CS	Stage	2	6608	3304	58.035	$\mathbf{4.04\times10^{-10}}$
	Diet	1	175	175	3.08	0.0915
	Stage×Diet	2	43	22	0.382	0.6866
	Residual	25	1423	57		
ETS	Stage	2	12.7	6.362	32.943	$\mathbf{5.70\times10^{-8}}$
	Diet	1	0.47	0.465	2.409	0.1322
	Stage×Diet	2	4.06	2.031	10.515	**0.0004**
	Residual	27	5.22	0.193		
PK:CS	Stage	2	0.42	0.2094	14.65	$\mathbf{6.15\times10^{-5}}$
	Diet	1	0.08	0.0818	5.725	**0.0246**
	Stage×Diet	2	0.06	0.0312	2.184	0.1336
	Residual	25	0.36	0.0142		
ETS:CS	Stage	2	0.01	0.0026	1.992	0.1567
	Diet	1	0	0.0036	2.672	0.1141
	Stage×Diet	2	0.03	0.0129	9.61	**0.0007**
	Residual	26	0.04	0.0013		

Supplementary Table S6. Two-way ANOVA evaluating the effect of the bacterial challenge with *V. splendidus* VPAP18 and the dietary treatment on the PK, CS and ETS enzyme activities and the PK:CS and ETS:CS ratios in larvae and early juveniles of the scallop *A. purpuratus*. Significant differences (P< 0.05) are in bold**.**

Parameter	Stage	Source	DF	SS	MS	F	*P*
PK	Veliger	Challenge	1	51.47	51.47	2.848	0.11
		Diet	1	18.94	18.94	1.048	0.32
		Challenge×Diet	1	4.64	4.64	0.257	0.619
		Residual	17	307.23	18.07		
	Pediveliger	Challenge	1	4.93	4.93	0.407	0.535
		Diet	1	46.78	46.78	3.868	0.072
		Challenge×Diet	1	18.23	18.23	1.507	0.243
		Residual	12	145.12	12.09		
	Juvenile	Challenge	1	12.27	12.27	2.769	0.112
		Diet	1	11.77	11.766	2.655	0.119
		Challenge×Diet	1	21.9	21.902	4.943	**0.038**
		Residual	19	84.19	4.431		
CS	Veliger	Challenge	1	0.09	0.087	0.161	0.69
		Diet	1	1.07	1.065	1.974	0.165
		Challenge×Diet	1	0.24	0.239	0.444	0.508
		Residual	60	32.39	0.539		
	Pediveliger	Challenge	1	1.707	1.707	6.345	**0.024**
		Diet	1	1.398	1.397	5.194	**0.038**
		Challenge×Diet	1	1.78	1.779	6.614	**0.022**
		Residual	14	3.767	0.269		
	Juvenil	Challenge	1	0.033	0.032	0.175	0.68
		Diet	1	0.091	0.091	0.492	0.491
		Challenge×Diet	1	0	0.0004	0.002	0.964
		Residual	20	3.713	0.185		
ETS	Veliger	Challenge	1	0.011	0.011	0.365	0.553
		Diet	1	0.111	0.111	3.628	0.072
		Challenge×Diet	1	0.028	0.028	0.928	0.348
		Residual	18	0.551	0.030		
	Pediveliger	Challenge	1	0.306	0.306	8.476	**0.010**
		Diet	1	0.961	0.961	26.6	$\mathbf{9.5\times10^{-5}}$

		Challenge×Diet	1	0.007	0.007	0.202	0.659
		Residual	16	0.578	0.036		
	Juvenile	Challenge	1	0.005	0.005	0.247	0.624
		Diet	1	0.139	0.139	6.327	**0.020**
		Challenge×Diet	1	0.038	0.038	1.733	0.202
		Residual	20	0.440	0.022		
PK:CS	Veliger	Challenge	1	0	0.000005	0	0.984
		Diet	1	0.00515	0.005147	0.439	0.516
		Challenge×Diet	1	0.01003	0.010034	0.857	0.368
		Residual	17	0.19912	0.011713		
	Pediveliger	Challenge	1	0.032	0.032	3.55	0.086
		Diet	1	0.106	0.106	11.5	**0.006**
		Challenge×Diet	1	0.012	0.012	1.308	0.277
		Residual	11	0.101	0.009		
	Juvenile	Challenge	1	0.079	0.079	0.372	0.549
		Diet	1	0.115	0.114	0.537	0.473
		Challenge×Diet	1	0.456	0.455	2.135	0.16
		Residual	19	4.056	0.213		
ETS:CS	Veliger	Challenge	1	0.004	0.004	0.066	0.800
		Diet	1	0.0004	0.0004	0.005	0.944
		Challenge×Diet	1	0.863	0.863	12.55	**0.002**
		Residual	16	1.101	0.068		
	Pediveliger	Challenge	1	1.802	1.802	15.73	**0.001**
		Diet	1	3.269	3.269	28.52	**0.0001**
		Challenge×Diet	1	0.052	0.052	0.457	0.509
		Residual	14	1.604	0.115		
	Juvenile	Challenge	1	0.015	0.015	0.032	0.860
		Diet	1	2.468	2.467	5.214	**0.033**
		Challenge×Diet	1	0.597	0.596	1.261	0.274
		Residual	20	9.466	0.473		

Supplementary Table S7. Two-way ANOVA evaluating the effect of diet and bacterial challenge on the respiration rate (nmol O2·h^{-1}·ind^{-1}) in veliger larvae of the scallop *A. purpuratus*.

Source	DF	SS	MS	F	*P*
Diet	1	0.0005265	0.000526	54,84	**<0,0001**
Challenge	1	0.008203	0.008203	854,5	**<0,0001**
Diet × Challenge	1	0.0005265	0.000526	54,84	**<0,0001**
Residual	20	0.000192	0.000009		

Supplementary Table S8. Two-way ANOVA evaluating the effect of ontogenic stage on the basal relative mARN expression of *ApTLR, ApIkB, ApBD1, ApLBP/BPI* and *ApGLys* in larvae and early juveniles of the scallop *A. purpuratus*. Significant differences (P< 0.05) are in bold.

Gene	Source	DF	SS	MS	F	*P*
ApTLR	Stage	2	9,769	4,884	50,798	**3,96E-08**
	Diet	1	2,501	2,501	26,01	**7,48E-05**
	Stage×Diet	2	0,049	0,024	0,255	0,778
	Residual	18	1,731	0,096		
ApIkB	Stage	2	7.147	3.573	89.31	**2.18E-10**
	Diet	1	2.865	2.865	71.59	**7.22E-08**
	Stage×Diet	2	1.478	0.739	18.48	**3.50E-05**
	Residual	19	0.76	0.04		
ApBD1	Stage	2	21.655	10.827	138.356	**8.10E-13**
	Diet	1	0.609	0.609	7.778	**0.011**
	Stage×Diet	2	1.649	0.824	10.534	**0.000679**
	Residual	21	1.643	0.078		
ApLBP/BPI	Stage	2	0.4739	0.23694	27.348	**1.08E-06**
	Diet	1	0.0009	0.00094	0.109	0.745
	Stage×Diet	2	0.0082	0.00409	0.472	0.63
	Residual	22	0.1906	0.00866		
ApGLys	Stage	2	2.6679	1.334	13.436	**0.000154**
	Diet	1	0.6401	0.6401	6.447	**0.018693**
	Stage×Diet	2	0.1127	0.0564	0.568	0.574901
	Residual	22	2.1842	0.0993		

Supplementary Table S9. Two-way ANOVA evaluating the effect of dietary treatment and bacterial exposure to *V. splendidus* VPAP18 on the relative expression of immune-related genes (*ApTLR, ApIkB, ApBD1, ApLBP/BPI1* and *ApGLys*) in larvae and early juveniles of the scallop *A. purpuratus*. Significant differences (P< 0.05) are in bold. The analysis type depended on the distribution of raw data.

Gene	Stage	Source	DF	SS	MS	F	*P*
ApTLR	Veliger	Diet	1	0,101	0,1009	0,302	0.589
		Challenge	1	0,857	0,8566	2,566	0.127
		Diet × Challenge	1	1,733	1,7326	5,191	**0.036**
		Residual	17	5,674	0,3338		
	Pediveliger	Diet	1	0.5758	0.5758	34.714	**7.34E-05**
		Challenge	1	0.3438	0.3438	20.727	**0.000663**
		Diet × Challenge	1	0.054	0.054	3.253	0.09642
		Residual	12	0.199	0.0166		
	Early juvenile	Diet	1	0.7897	0.7897	11.501	**0.0116**
		Challenge	1	0.0692	0.0692	1.007	0.349
		Diet × Challenge	1	0.0046	0.0046	0.067	0.8035
		Residual	7	0.4806	0.0687		
ApIkB	Veliger	Diet	1	9.039	9.039	140.388	**6.19E-10**
		Challenge	1	0.099	0.099	1.544	0.23
		Diet × Challenge	1	0.046	0.046	0.716	0.408
		Residual	18	1.159	0.064		
	Pediveliger	Diet	1	0.0247	0.0247	0.288	0.60207
		Challenge	1	0.115	0.115	1.343	0.27101
		Diet × Challenge	1	0.8994	0.8994	10.509	**0.00785**
		Residual	11	0.9414	0.0856		
	Early juvenile	Diet	1	0.01626	0.01626	3.232	0.1153
		Challenge	1	0.03317	0.03317	6.594	**0.0371**
		Diet × Challenge	1	0.00914	0.00914	1.817	0.2196
		Residual	7	0.03521	0.00503		
ApBD1	Veliger	Diet	1	0.3057	0.30566	2.13	0.16
		Challenge	1	0.0389	0.03891	0.271	0.608
		Diet × Challenge	1	0.1367	0.1367	0.953	0.341
		Residual	20	2.8697	0.14349		
	Pediveliger	Diet	1	1.4134	1.4134	22.326	**0.000493**
		Challenge	1	2.9165	2.9165	46.07	**1.94E-05**
		Diet × Challenge	1	0.0445	0.0445	0.703	0.41824
		Residual	12	0.7597	0.0633		

	Juvenil	Diet	1	2.7398	2.7398	23.744	**0.00181**
		Challenge	1	0.6275	0.6275	5.438	0.05246
		Diet × Challenge	1	0.03	0.03	0.26	0.62587
		Residual	7	0.8077	0.1154		
ApLBP/BPI1	Veliger	Diet	1	0.0734	0.07336	0.75	0.397
		Challenge	1	0.0215	0.02146	0.219	0.645
		Diet × Challenge	1	0.0106	0.01059	0.108	0.746
		Residual	20	1.957	0.09785		
	Pediveliger	Diet	1	0.03437	0.03437	17.217	**0.001**
		Challenge	1	0.04681	0.04681	23.454	**0.0003**
		Diet × Challenge	1	0.00674	0.00674	3.376	0.087
		Residual	14	0.02794	0.002		
	Early juvenile	Diet	1	0.00142	0.00142	0.155	0.706
		Challenge	1	0.00188	0.001879	0.205	0.665
		Diet × Challenge	1	0.00054	0.00054	0.059	0.815
		Residual	7	0.06424	0.009177		
ApGLys	Veliger	Diet	1	0.0138	0.01377	0.123	0.729
		Challenge	1	0.002	0.00202	0.018	0.894
		Diet × Challenge	1	0.4310	0.4310	4.969	**0.032**
		Residual	20	2.2337	0.11169		
	Pediveliger	Diet	1	0.02691	0.02691	2.887	0.111
		Challenge	1	0.01741	0.01741	1.868	0.193
		Diet × Challenge	1	0.03324	0.03324	3.565	0.074
		Residual	14	0.13051	0.00932		
	Early juvenile	Diet	1	0.3783	0.3783	35.848	**0.0005**
		Challenge	1	0.0022	0.0022	0.209	0.661
		Diet × Challenge	1	0.0016	0.0016	0.148	0.712
		Residual	7	0.0739	0.0106		

Supplementary Table S10. Two-way ANOVA evaluating the effect of dietary treatment and bacterial exposure to *V. splendidus* VPAP18 on total *Vibrio* growth in veliger larvae of the scallop *A. purpuratus.* Significant differences (P< 0.05) are in bold.

**Source**	**DF**	**SS**	**MS**	**F**	***P***
Diet	1	10667	10667	18.284	**0.00523**
Challenge	1	8561	8561	14.675	**0.00865**
Diet × Challenge	1	1620	1620	2.777	0.14665
Residual	6	3500	583		

Supplementary Table S11. Two-way ANOVA evaluating the effect of farming time and dietary treatment on *A. purpuratus* larval growth/length and survival. Significant differences (P< 0.05) are in bold.

**Parameter**	**Source**	**DF**	**SS**	**MS**	**F**	**P**
Growth/	Farming time	5	5201667	1040333	10403	**<0.0001**
First spawn	Diet	1	133333	133333	1333	**<0.0001**
	Time × Diet	5	174667	34933	349	**<0.0001**
	Residual	108	10800	100		
Growth/	Farming time	4	133276	33319	433	**<0.0001**
Second spawn	Diet	1	3900	3900	50.75	**<0.0001**
	Time × Diet	4	1594	398	5.186	**0.0005**
	Residual	230	17675	76.85		
Survival	Farming time	5	70627	14125	2724	**<0.0001**
	Diet	1	7695	7695	1484	**<0.0001**
	Time × Diet	5	2250	450	86.78	**<0.0001**
	Residual	250	1296	5.185		
